# Randomized Clinical Outcome Trials in Hypertension

**DOI:** 10.1161/HYPERTENSIONAHA.123.21725

**Published:** 2023-10-05

**Authors:** Giuseppe Mancia, Sverre E. Kjeldsen

**Affiliations:** University of Milan-Bicocca, Milan, Italy (G.M.).; University of Oslo, Institute of Clinical Medicine, Medical Faculty, Oslo, Norway (S.E.K.).; Departments of Cardiology and Nephrology, Oslo University Hospital, Ullevål, Norway (S.E.K.).

**Keywords:** blood pressure, cardiovascular diseases, clinical trials, drug therapy

Outcome-based randomized trials on the treatment of hypertension have preceded by several years trials on the treatment of other major cardiovascular risk factors, such as dyslipidemia and diabetes. The first trials were conducted in the Veterans Administration system in the United States on some hundred males with severe hypertension and high cardiovascular risk and published in 1967^1^ and 1970,^2^ respectively for the very high and high-to-medium blood pressure (BP) strata. The results showed that treatment with the drugs available at that time (mainly diuretics, central agents, and hydralazine) was accompanied over about 4 years by a major reduction of cardiovascular events compared with patients who had been randomized to placebo, thereby providing evidence that the hypertension-related cardiovascular risk is not irreversible, but it can be reduced by a BP-lowering intervention. In the subsequent 20 years, this conclusion was further tested by a dozen additional randomized clinical trials (RCTs) that recruited patients of both sexes, milder hypertension grades, or an age above 60 or 65 years, using antihypertensive drugs in part or completely different from those of the original trials. The protective nature of BP-lowering interventions was confirmed, and this was the case also for the large magnitude of the protective effect. In a meta-analysis of randomized outcome trials on about 37 000 patients published in 1990, systolic BP (SBP) and diastolic BP (DBP) reductions of 10 and 5 mm Hg, respectively, were reported to reduce stroke by about 42%, coronary disease by about 14%, and cardiovascular mortality by about 20%.^3^

## SUBSEQUENT TRIAL ACHIEVEMENTS IN PATIENTS WITH HYPERTENSION

The demonstration of the protective effect of antihypertensive treatment by earlier trials favored in the following decades the design and conduction of many more trials that allowed the effect of BP reduction on outcomes to be studied in several hundred thousand patients characterized by widely different demographic and clinical conditions. While confirming the protective nature of BP-lowering treatment, these trials (together with their subgroups analyses and meta-analyses) have enormously increased previous knowledge, with a crucial contribution to treatment guidelines in this medical area.^4^ A handful of trials (MAPHY [Metoprolol Arm of the Heart Attack Primary Prevention in Hypertension], HAPPHY [Heart Attack Primary Prevention in Hypertension study], Medical Research Council I, Medical Research Council II, International Prospective Primary Prevention Study in Hypertension) compared around 1990 outcomes on diuretics of the thiazide type with various beta-blockers without findings of major differences at least not in the primary end point. Further, a handful of trials in the mid-later 1990s compared ACE (angiotensin-converting enzyme) inhibitors, calcium channel blockers, and the alpha-blocker doxazosin with thiazide diuretics, thiazide/beta-blocker combination or chlorthalidone (Table) and found no differences in the primary end points, which were cardiovascular mortality, a composite of stroke, myocardial infarction and cardiovascular mortality, or incident myocardial infarction and fatal coronary disease. Already, in the year 2000, there was clear indication suggesting that BP-lowering treatment with these rather well-tolerated 5 main drug classes or their combinations was similarly effective in preventing hypertensive complications.^5^

**Table.**
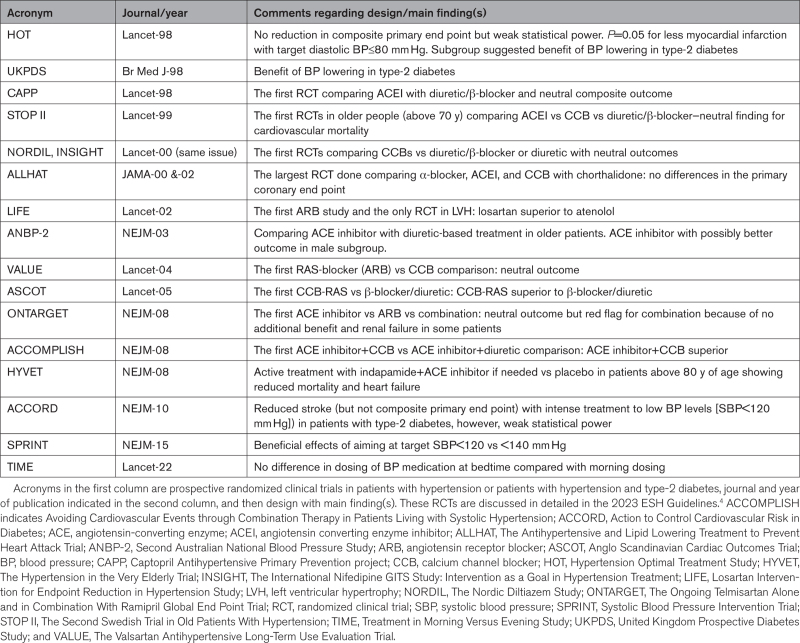
Selected Landmark RCTs in Patients With Hypertension Discussed in Detail in Mancia et al^4^

## ADDITIONAL PROTECTIVE EFFECTS OF ANTIHYPERTENSIVE TREATMENT

Trials performed in and after the nineties confirmed the suggestion advanced by the earliest trials that in hypertensive patients, BP reduction substantially reduced incident heart failure, the major size of the reduction (about −50% risk) implying that the 2 most common heart failure phenotypes (heart failure with reduced and preserved ejection fraction) are both beneficially affected. Evidence has also been obtained that the benefits of antihypertensive treatment (1) extend to all-cause mortality, by about 10%,^3,4^ an effect not clearly visible in the earliest trials that had implications for public health policies; (2) reduced the development of end-stage kidney disease and the related risk to go on dialysis, kidney transplantation, and cardiovascular nonlethal and lethal events in patients with both diabetic or nondiabetic nephropathy; (3) protects against both ischemic (thrombotic and embolic) and hemorrhagic strokes. Despite some data heterogeneity, results of current trials also support the conclusion that the effect of BP-lowering treatment on the brain includes a reduction of cognitive dysfunction and dementia (conditions for which hypertension has been shown to be an important risk factor), presumably via protection against diffuse damage of small cerebral vessels and subsequent white matter lesions or prevention of lacunar strokes as recently reviewed in detail in the 2023 European Society of Hypertension guidelines.^4^

## ANTIHYPERTENSIVE TREATMENT AND DEMOGRAPHIC DIFFERENCES

Although never addressed by specific antihypertensive treatment trials, in most trials, women have been included as frequently as men, leading to large subgroup analyses that leave no doubt as to the cardiovascular and renal protective effects of antihypertensive treatment in both sexes. Based on few specific trials and subgroup analyses, it is also clear that the protective effect of BP-lowering interventions includes Whites, Blacks, and Asian patients, thereby extending to major ethnicities. Trials recruiting patients aged 70 (STOP I and II [Swedish Trial in Old Patients with hypertension I and II]) or even 80 (HYVET [Hypertension in the Very Elderly Trial]) years or above (Table) have allowed to document that the BP-lowering interventions reduce cardiovascular events also in very old patients, supporting the now generalized recommendation of major guidelines that it is mandatory to control an elevated BP under these demographic circumstances.^4^ Dedicated trials performed in United States, Europe, and Asia (SHEP [Systolic Hypertension in the Elderly Program], SYST-EUR [Systolic Hypertension in Europe Study], SYST-CHINA [Systolic Hypertension in China Study]) have also consistently shown that antihypertensive treatment reduces cardiovascular outcomes in isolated systolic hypertension (ISH) which has a high prevalence in the old ages and an especially high cardiovascular risk. This is an important extension of available knowledge because the high SBP and normal or low DBP typical of this condition reflect a mechanistic difference of ISH from a systolic-diastolic BP elevation, that is, a marked stiffening of large arteries rather than or beyond an increase of systemic vascular resistance.

## ANTIHYPERTENSIVE TREATMENT AND COMORBIDITIES

Dedicated trials or subanalyses of large trial subgroups show that antihypertensive treatment is beneficial not only in the absence but also in the association with comorbidities, which happens in large number of patients due to the extremely high prevalence of hypertension in the population as well as to the adverse interaction of an elevated BP with many other pathophysiological factors. To quote some examples, reduction of an elevated BP reduces cardiovascular events in both the primary and the secondary prevention setting, that is, in patients with no or previous cardiovascular events such as myocardial infarction or stroke. Antihypertensive treatment protects against stroke, heart failure, or other outcomes in hypertensive patients with atrial fibrillation, in which the BP reduction also lowers the risk of intracranial bleeding that accompanies in these patients’ use of anticoagulants to reduce the increased risk of embolic stroke.^4^ Hypertensive dyslipidemic or type-2 diabetic patients exhibit a reduction of cardiovascular outcomes when BP-lowering treatment is implemented, and this is the case also in association with lipid lowering or antidiabetic drugs. Indeed, therapeutic control of additional cardiovascular risk factor does not seem to lower the protective effect of reducing an elevated BP, suggesting that its effectiveness is established independently on other risk attenuating strategies.^4^ Although no trial has specifically focused on obese hypertensive patients, obesity or overweight is so common in individuals with BP elevation as to make the related trial database strikingly large. This database shows that, like normal weight patients, obese hypertensive patients have less cardiovascular events if BP is reduced.^4^ At variance from hypertensive diabetic or dyslipidemic patients, no trial to date has explored the effect of BP-lowering drugs on top of pharmacological or surgical treatment of obesity.

## CARDIOVASCULAR AND RENAL PROTECTION BY DIFFERENT ANTIHYPERTENSIVE DRUGS AND TREATMENT STRATEGIES

During the 50 years of the antihypertensive trial era, a major issue has been whether for a similar BP reduction, the protective effect of antihypertensive treatment differs between different antihypertensive drugs or treatment strategies. This combined the commercial interest of some pharmaceutical companies, which were willing to sponsor investigator-initiated RCTs to obtain evidence in favor of their own drugs, with the research goals to (1) identify which strategies might be more effective for achieving BP reduction, (2) whether some drugs might have BP-independent protective effects that could increase the patients’ overall protection at a given on-treatment BP level, and (3) which demographic and clinical subgroups of patients might exhibit the above two advantages. The large number of trials that have compared different antihypertensive treatments (Table) have not provided entirely unequivocal findings and thus the questions whether at similar on-treatment BP levels some drugs or treatment strategies are more protective than others in all or some patients are still matter for research. However, few undisputable findings have emerged and have made their way into recommendations by guidelines.^4^ For example, it is clear from comparison (but also from placebo-controlled) trials that the best treatment strategy to reduce and control an elevated BP is to combine drugs with different mechanisms of action. It is also clear that some antihypertensive drugs may have BP-independent protective properties, two relevant examples being the greater kidney protection associated with use of blockers of the renin-angiotensin system in diabetic (RENAAL [Reduction in Endpoints in NIDDM with the Angiotensin II Antagonist Losartan], IDNT [Irbesartan Diabetic Nephropathy Trial]) or nondiabetic nephropathy and the regression of left ventricular hypertrophy with subsequent cardiovascular protection (Table). However, the target BP to achieve optimal kidney and heart protection are yet to be settled though probably at not much (or no) variance from the target BP in the general hypertensive population (see below).^4^

More recently, another example has emerged from the trials on the renal and cardiovascular protective effects of sodium-glucose cotransporter 2 inhibitors in both diabetic and nondiabetic patients, which can now be convincingly ascribed to their intrinsic rather than BP-lowering properties. However, as far as the cardiovascular effects of treatment in the general hypertensive population is concerned, the strongest message disseminated by large meta-analyses of randomized trials has been that the benefits of treatment mainly depend on treatment-induced BP reductions or on-treatment absolute values because in treated patients these effects linearly relate with outcomes, regardless the type of treatment.^4^ In this context, the huge amount of evidence produced by the 50-year trial era has led to a simple message, that is, what matters for patient’s protection by treatment is BP control much more than the drug(s) through which control is obtained. Yet, search for BP-independent protective properties of drugs to be used in hypertension should not be abandoned because this is one of the possibilities to reduce residual cardiovascular risk, which is not normalized by even optimal BP control.^4^

## BP THRESHOLD AND TARGET FOR TREATMENT

Correction of a medically inappropriate lifestyle should be considered at any BP level because it can favorably modify cardiovascular risk and prevent or reduce new-onset hypertension with virtually no inconveniences.^4^ Whether drug treatment of people with high normal BP or prehypertension may prevent future hypertension has also been suggested but may need confirmation, to be focused on the balance between prevention of future hypertension and drug-dependent side effects or other inconveniences.^6^ In contrast, due to side effects, the use of antihypertensive drugs is recommended only at BP levels at which there is evidence that a BP reduction is accompanied by a protective effect that outbalances the potential drug-related problems. In most hypertension guidelines including European Society of Hypertension guidelines,^4^ these threshold BP levels for treatment have been located at a SBP≥140 mm Hg or a DBP≥90 mm Hg because these are the entry BP criteria of most trials that have documented a reduction of cardiovascular outcomes by BP-lowering interventions compared with a placebo or control group. Trial-based evidence, however, also suggests that in some clinically important subgroups, different BP thresholds may have to be taken into consideration. This is the case, for example, for patients with ISH or aged ≥80 years in whom the strongest evidence (from dedicated trials) suggests drug treatment initiation at SBP values ≥160 mm Hg,^7^ but lower thresholds are left open based on subgroup analysis of several trials.^8^ In an opposite direction, it is also the case for patients with previous cardiovascular events in which a trial meta-analysis^9^ has shown a protective effect of BP reduction when initial BP is in the high normal BP range (130–139/85–89 mm Hg).

Outcome-based trials are also the source of information on the target BP to be reached with treatment, identified as the on-treatment BP at which the treatment-dependent protection is maximized. Available results are not entirely unambiguous due to differences in the trial design, the approach to BP measurements, the heterogeneity of the patients’ clinical and demographic characteristics, and the type of data analysis. Nevertheless, a univocal trial-based message^4^ is that in most hypertensive patients, reducing SBP to <140 mm Hg and DBP to <80 mm Hg accounts for a substantial fraction of the BP-dependent protection of antihypertensive treatment, thereby representing the must target to be considered for a wide range of hypertensive individuals. Over the same age range, however, another trial-based message^4^ is that a further reduction of SBP to 130 mm Hg or less may be accompanied by a further benefit and should thus be considered if treatment is well tolerated by the patients. As for the BP threshold for treatment, this may not be true for all patients, and indeed trial data suggest that in people with ISH, a very old age or other conditions, more conservative BP targets may be appropriate. Thus, trial data on different BP threshold and target for treatment exemplify the contribution of trial research to individualization of treatment, a goal that will probably be more consistently pursued by future trials that will recruit more heterogeneous patient populations to pragmatically be closer to real life.

## INTENSIVE LOWERING OF BP AND RESISTANT HYPERTENSION

More intensive lowering of BP has been a major issue for antihypertensive treatment trials since the late 1980s, based on the opposite message from epidemiological data and pathophysiological considerations. By showing that the risk of hypertension decreases progressively down to about 110/70 mm Hg, epidemiological data carried the message that intensive antihypertensive treatment might enhance patient protection while pathophysiology warned that, in hypertensive patients with structural vascular alterations and possible damage of blood flow autoregulation, marked BP reductions might lead to organ under-perfusion and even an increased risk, that is, a J-curve phenomenon.^4^ Few major RCTs (HOT [Hypertension Optimal Treatment Study], ACCORD [Action to Control Cardiovascular Risk in Diabetes], SPRINT [Systolic Blood Pressure Intervention Trial]) have addressed this issue with the use of automated devices for standardization and accuracy of BP measurements. The HOT and the ACCORD (in type-2 diabetic patients) did not show differences for the primary end point aiming at DBP<80 mm Hg and SBP<120 mm Hg, respectively (actual on-treatment values averaged 81, 83, and 85 mm Hg in HOT and 119 versus 134 mm Hg in ACCORD). This may have happened because of limiting factors such as small between-group DBP differences (HOT), low statistical power or inconveniences due to the factorial design of both trials, such as patient randomization also to acetylsalicylic acid in HOT^10^ and to statin and glucose-lowering drugs in ACCORD.^11^ Indeed, in ACCORD, removal of the increased incidence of events with intensive blood glucose lowering made the benefit of intensive BP reductions more clear.^12^ Furthermore, in both trials, more clear benefits were seen by secondary end points or subgroup analyses (Table).

In the SPRINT Study, the primary publication^13^ reported an on-treatment BP of 121.5 mm Hg in the intensive-treatment group and 134.6 mm Hg in the standard-treatment group, as a result of aiming at target SBP <120 versus <140 mm Hg (Table). The intervention was stopped early after a median follow-up of 3.26 years owing to a significantly lower rate of the primary composite outcome in the intensive-treatment group than in the standard-treatment group (1.65 versus 2.19% per year), a finding that was confirmed in a large subgroup of patients aged ≥75 years (average, 80 years).^14^ These results have played a major role in the recommendation of the latest US guidelines to lower SBP to <130 rather than <140 mm Hg in virtually the entire hypertensive population. They have also influenced the choice of a similar target in the 2023 European Society of Hypertension guidelines,^4^ although with the exclusion of some subgroups of patients and caveats due to the heterogeneous approach of SPRINT to BP measurements (attended and unattended BP) as well as the marked increase of serious side effects at SBP values of <130 mm Hg.

Resistant hypertension, defined as the lack of BP control despite full doses of 3 antihypertension drugs including a diuretic, has attracted major attention in recent years because, although relatively rare compared with the overall treated hypertensive population (<5%–10%), it is characterized by a marked increase of cardiovascular risk.^4^ No outcome trial has ever been performed in this condition while recent randomized trials have documented that, in patients with resistant hypertension who completed 3 treatment cycles with different add-on medications and placebo, spironolactone was the most effective added BP-lowering drug, although with an increased risk of hyperkalemia despite its minimization by the choice of excluding patients with advanced kidney damage.^15^ Recent randomized trials performed in United States and Europe have also shown the BP-lowering effect of renal denervation compared with patients with sham-denervation.^4^ However, the limited magnitude of the BP-lowering effect as well as the absence of outcome data make resistant hypertension an area in need of further trials, possibly considering novel agents that have shown both the ability to lower BP and to exert renal and cardiac protective effects.^4^

## OUTCOME TRIAL LIMITATIONS

Despite their impressive results, evidence provided by outcome-based randomized trials in hypertension shows important limitations and gaps that leave some clinical areas unaddressed and perhaps un-addressable by the randomized trial approach. An important limitation is that trials are conducted under conditions that are medically far better (lower therapeutic inertia, higher adherence, better physicians’ expertise, lower number of medical errors, etc) than those characterizing clinical practice.^4^ This means that their results reflect the potential of antihypertensive treatment efficacy but cannot provide a precise picture of how much of the potential is translated into clinical practice, that is, how much efficacy translates into effectiveness. A paradigmatic example is the rate of BP control, which is strikingly greater in trials than at the level of the hypertensive population.

Other limitations are that for a variety of reasons important aspects of antihypertensive treatment have not been addressed and several patient categories have never been included in antihypertensive treatment trials. For example, the DBP to aim at during antihypertensive treatment of old patients with an exclusive or prevalent SBP elevation has never been established by trials, leaving unanswered the question of whether in this common clinical condition too low on-treatment DBP values impair organ perfusion and attenuate, nullify, or reverse the benefits of SBP reductions. Another example is that for logistic reasons all major trials did not measure out-of-office BP except in small nonrandomized subgroups, thereby failing to provide any information on whether guiding treatment by ambulatory or home BP results in greater patient protection than guiding treatment by office BP.^16^ Finally, in many patients’ categories, there is no information on whether BP reduction is accompanied by any benefit at all. One example is frail patients although some evidence on the protective effect of antihypertensive treatment in these patients has been recently reported in SPRINT^14^ and large real-life databases^17^; but, patients with <40 years of age, patients with isolated diastolic hypertension, young patients with ISH, and patients with BP phenotypes such as white coat hypertension, masked hypertension, nocturnal hypertension, or abnormal BP reductions have never been addressed by randomized trials designed to determine whether antihypertensive treatment is associated with reduction in their cardiovascular risk.^4^

This makes it necessary for guidelines to extrapolate treatment recommendations from evidence obtained in other patients and conditions, with the inevitable risk of errors. This is unfortunate also because of the high prevalence of some of these categories. For example, white coat hypertension may account for at least one-third of all hypertensive patients while masked hypertension has been reported in about 1 out of 7 individuals with a normal office BP. While some of these gaps will hopefully be filled by future trials, in other instances, research approaches other than randomized outcome trials will be necessary. This is the case for the benefits of treatment in young hypertensive patients in whom the limited number of outcomes traditionally used in trials suggests that other treatment-related benefits should be considered, an obvious choice being treatment-dependent changes in prognostically validated subclinical organ damage.^4^ This has a strong rationale in young individuals in whom the goal of treatment is mainly to oppose the silent progression of organ damage that will lead to a high cardiovascular risk and perhaps to outcomes years later.

## FLAWED RESEARCH REGARDING TRIALS IN HYPERTENSION

Safety characteristics of RCTs in hypertension have been (1) the large organizations being created for doing the trials, (2) the multicenter double-blinded design, (3) public approval, and (4) audit and monitoring of details related to every patient mainly source data verification. Adherence to drug treatment has also been monitored, mostly by pill count. However, patients have not necessarily been titrated to target BPs as sign of investigator (physician) inertia^18^ and RCT conducts may dampen the beneficial effects of treatment because of the intention-to-treat statistics. Other serious flaws have been documented.^19^ Whereas source data verification has been a key element in the RCTs discussed above and in numerous other RCTs, lack of such verifications by the investigators has led to retraction of RCTs when questions have been raised.^19^ Responsibilities also rest with reviewers and editors who may accept and publish flawed RCTs. A serious ongoing situation has not yet been definitively resolved^19^: recent RCT data^20^ suggest a study outcome has been fabricated by the authors as previously discussed.^19^ At the end, much responsibility comes with meta-analyses, which may do fundamental mistakes as explained in an essentially undisputed criticism.^21^

## PERSPECTIVES

Hypertension is consistently ranked by the World Health Organization as the No. 1 cause of morbidity and mortality worldwide.^4^ This reflects the limited control of an elevated BP in the real-life population because evidence from numerous RCTs performed through 50 years has un-disputably proven that drug treatment is effective in both lowering BP and preventing the multiple hypertension-related complications from the brain, the heart, the kidneys, and the large arteries. Trials have further shown that (1) the overall RCT benefits are mostly related to the size of BP lowering or the achieved average BPs, (2) renin-angiotensin-aldosterone system-inhibitors, calcium channel blockers, beta-blockers, and diuretics are the main classes of antihypertensive drugs through which effective BP reduction and outcome protection can be achieved, and (3) combinations of the above drug classes are the most effective BP-lowering strategies, their use guaranteeing BP control in most hypertensive individuals, with an adherence to treatment that is favored by their availability in a single pill. Despite these extraordinary achievements, there are still important limitations in the knowledge of the relationship between treatment-dependent BP reduction and outcome, including that the effects of treatment remain unknown in major subgroups of hypertension, some of them (white coat hypertension and masked hypertension) representing large strata of the hypertensive population. Considering its large prevalence (about half of the adult and old population) and prognostic impact, there is the need for future RCTs on many still incompletely clear or unaddressed aspects of hypertension. Research should extend to the collection of real-life data to also improve measurement and knowledge of issues such as poor adherence to drug treatment and physician inertia, that is, the factors that are fundamentally responsible for poor BP control and adverse cardiovascular impact of high BP in medical practice.

## ARTICLE INFORMATION

### Sources of Funding

None.

### Disclosures

G. Mancia reports honoraria from Astra Zeneca, Boehringer Ingelheim, Daiichi Sankyo, Medtronic, Menarini, Merck Healthcare KGaA, Novartis, Recordati, Sandoz, Sanofi, and Servier. S.E. Kjeldsen reports lecture honoraria from Getz, J.B. Pharma, Merck Healthcare KGaA, Vector-Intas, and Zydus.
